# Association Between Decline in Cognitive Processing Speed and CSF Amyloid‐β 42/40 in Cognitively Healthy Older Adults Aged 70, Followed over 5 to 7 Years: Data from the Gothenburg H70 Birth Cohort Study

**DOI:** 10.1002/alz70857_105138

**Published:** 2025-12-25

**Authors:** Kelly Rombauts, Johan Skoog, Tobias Skillbäck, Anna Dittrich, Henrik Zetterberg, Kaj Blennow, Ingmar Skoog, Silke Kern

**Affiliations:** ^1^ Institute of Neuroscience and Physiology, Sahlgrenska Academy, University of Gothenburg, Gothenburg, Sweden; ^2^ Neuropsychiatric Epidemiology Unit, Sahlgrenska Academy, University of Gothenburg, Mölndal, Sweden; ^3^ Region Västra Götaland, Sahlgrenska University Hospital, Department of Neuropsychiatry, Mölndal, Sweden; ^4^ Clinical Neurochemistry Laboratory, Sahlgrenska University Hospital, Mölndal, Västra Götaland län, Sweden; ^5^ Hong Kong Center for Neurodegenerative Diseases, Hong Kong, Science Park, China; ^6^ UCL Institute of Neurology, Queen Square, London, United Kingdom; ^7^ Wisconsin Alzheimer's Disease Research Center, University of Wisconsin‐Madison, School of Medicine and Public Health, Madison, WI, USA; ^8^ UK Dementia Research Institute at UCL, London, United Kingdom; ^9^ Paris Brain Institute, ICM, Pitié‐Salpêtrière Hospital, Sorbonne University, Paris, France; ^10^ Neurodegenerative Disorder Research Center, Division of Life Sciences and Medicine, Institute on Aging and Brain Disorders, University of Science and Technology of China and First Affiliated Hospital of USTC, 合肥, 安徽, China; ^11^ Clinical Neurochemistry Laboratory, Sahlgrenska University Hospital, Mölndal, Sweden

## Abstract

**Background:**

Understanding early signs of cognitive decline in preclinical Alzheimer´s disease may open early intervention to slow down disease progression. We aimed to study the association between Amyloid‐β42/40 (Aβ) and cognitive decline, in a population‐based cohort of 70‐year‐olds followed over 5 to 7 years.

**Method:**

The baseline sample (*n* = 250) was drawn from the 2014 – 2016 assessments conducted within the H70 Gothenburg Birth Cohort Study (*n* = 1203, response rate=72%), Sweden. At age 70, 322 (26%) individuals consented to lumbar puncture. Participants with dementia (*n* = 5) or CDR score ≥ 1 (*n* = 1) were excluded. Participants who underwent cognitive examination at baseline and follow‐up (2019 – 2022) were included, resulting in a sample of 250 participants. Cerebrospinal fluid (CSF) levels of Aβ42, Aβ40, total tau (t‐tau), and phosphorylated tau (*p*‐tau) were measured. Six cognitive domains (memory, attention, executive function, verbal fluency, visuospatial abilities, and processing speed) were created after z‐standardizing raw scores from 11 neuropsychological tests. These were combined into a composite score. Multiple linear regression models were used to evaluate the associations between Aβ42/40 and change in composite score and cognitive domains, adjusting for age, sex, and years of education.

**Results:**

Of the 250 participants, 121 (48.4%) were female and the mean age at follow‐up was 76.06 (SD 0.48). At baseline, the prevalence of Aβ pathology (Aβ42/40 ≤0.082) was 25.9%, t‐tau (≥350 pg/mL) 29.2%, and *p*‐tau (≥80 pg/mL) 5.6%, as previously published in a partially overlapping subsample (Kern et al., 2018). Lower levels of Aβ42/40 were associated with a steeper decline in processing speed over the follow‐up period (β 0.123; 95% CI [0.018 – 1.042]). No significant associations were found between Aβ42/40 and changes in the other five cognitive domains or the composite score. Additionally, women showed a slower decline than men in the composite score (β 0.248; 95% CI [0.081 – 0.302]).

**Conclusion:**

Our results suggest that a decline in cognitive processing speed is associated with lower CSF Aβ42/40 before a decline in other cognitive domains appears. Additionally, our results indicate that females experience a slower rate of cognitive decline than males, between ages 70 and 76, independent of Aβ42/40.

